# Giant Benign Ovarian Serous Cystadenoma in a Postmenopausal Woman

**DOI:** 10.7759/cureus.108828

**Published:** 2026-05-14

**Authors:** George Mpourazanis, Apostolos Ntanasis, Magdalini Aliri, Elisavet Melissi, Georgia Christodoulou, Dimitrios Alefragkis, Zoi Anastasiadi, Savvas Tsigas, Panagiotis Oikonomou, Panagiotis Tsirkas, Aikaterini Ntaflou, Freideriki Steliou, Christos Akrivis

**Affiliations:** 1 Department of Obstetrics and Gynecology, General Hospital of G. Chatzikosta, Ioannina, GRC; 2 Department of Anesthesiology, General Hospital of G. Chatzikosta, Ioannina, GRC; 3 Second Department of Critical Care, Medical School, Attikon University Hospital, National and Kapodistrian University of Athens, Athens, GRC; 4 Department of Nursing, School of Health Sciences, National and Kapodistrian University of Athens, Athens, GRC; 5 Department of Radiology, General Hospital of G. Chatzikosta, Ioannina, GRC; 6 Department of Pathology, General Hospital of G. Chatzikosta, Ioannina, GRC

**Keywords:** ovarian cyst, ovarian cystadenoma, ovarian serous cystadenoma, postmenopausal woman, serous cystadenoma

## Abstract

Giant benign serous cystadenomas are ovarian tumors predominantly found in postmenopausal women, often presenting with nonspecific symptoms prior to diagnosis. This case study discusses an 80-year-old postmenopausal woman who experienced dyspnea and lower abdominal pain, leading to a contrast-enhanced abdominal computed tomography that revealed a well-defined giant cystic mass. The patient underwent a total hysterectomy with bilateral salpingo-oophorectomy (THBSO), with the excised specimen measuring 40 cm in diameter and weighing approximately 7.7 kg. Histopathological examination confirmed that cyst formation was lined by a single layer of columnar and cuboidal epithelium with fibrous stroma, identifying it as a benign serous cystadenoma. The patient had an uneventful postoperative recovery over 60 days. The uniqueness of this case lies in the patient's age and the atypical clinical presentation of the giant cystic mass. Additional clinical multicenter studies and molecular studies are needed to be done in the future in order to better understand the mechanism creating these tumors and tumorigenesis.

## Introduction

Based on the scientific literature, giant benign serous cystadenomas are ovarian tumors characterized by a solid surface and fluid, as noted in various scientific literature. These tumors can affect women of all age groups and are associated with significant mortality and morbidity.

Numerous studies identify giant ovarian cysts measuring above 10 cm in maximum diameter that extend beyond the umbilicus [1]. According to the etiology of ovarian serous cystadenomas, an experimental study using molecular genetic analysis demonstrated that these tumors' genes, BRAF or KRAS, appear to be polyclonal, and they do not contain mutations. Furthermore, the development of serous cystadenomas is linked to hyperplastic expansion from epithelial inclusions, where clonal/neoplastic transformation occurs in a subset [2]. 

Benign serous cystadenomas are classified based on the epithelium differentiation as serous, mucinous, endometrioid, clear cell, and urothelial tumors. These tumors are also classified into benign, borderline, and malignant tumors. Benign tumors are classified into three categories based on "components": cystadenoma, cystadenofibroma, and adenofibroma, with consideration given to the level of stratification and invasion. The occurrence rate is about 25% of all benign and 58% of all ovarian serous tumors [3-5]. Research indicates that 50% of serous tumors occur during the reproductive years, particularly before age 40. In premenopausal women, there is a 7-13% chance of a benign tumor becoming malignant, while in postmenopausal women, the incidence rises to 8-45% [6]. Giant ovarian serous cystadenomas are uncommon among elderly women worldwide. 

In this case study, an 80-year-old postmenopausal woman presented with chronic dyspnea and abdominal pain. Examination revealed a large mass indicating a gravid uterus of approximately 34 weeks, but a CT scan identified it as a massive ovarian serous cystadenoma. The patient underwent total abdominal hysterectomy with bilateral salpingo-oophorectomy (TAHBSO) as her treatment. The normal tumor markers within normal limits and the specific ovarian pathology highlight the rarity of this case, justifying its detailed discussion.

## Case presentation

An 80-year-old Caucasian postmenopausal patient presented to the gynecology outpatient clinic due to abdominal pain and discomfort that had persisted for several months. Additionally, she experienced dyspnea during the last month. Notably, she did not report any urinary or bowel symptoms, nor did she exhibit signs of weakness or anorexia during her consultation. Her medical family history reported no similar symptoms or any health issues. Her vital signs were stable, with a blood pressure of 115/75 mmHg, a temperature of 37°C, a pulse rate of 86 beats per minute, a respiratory rate of 16 breaths per minute, and an oxygen saturation level of 94%. Her body mass index (BMI) was calculated at 31 kg/m². Family medical history reported no breast or ovarian malignancy. The patient's medical history included diabetes mellitus type I, hypertension, dyslipidemia, and depression. She reported no allergies, smoking habits, or use of alcohol. The patient's daily medication included pantoprazole 40 mg, metformin 1000 mg, amlodipine 5 mg, paroxetine 20 mg, and atorvastatin calcium trihydrate, along with ezetimibe 10/20 mg. All medication was administered once a day.

**Figure 1:**
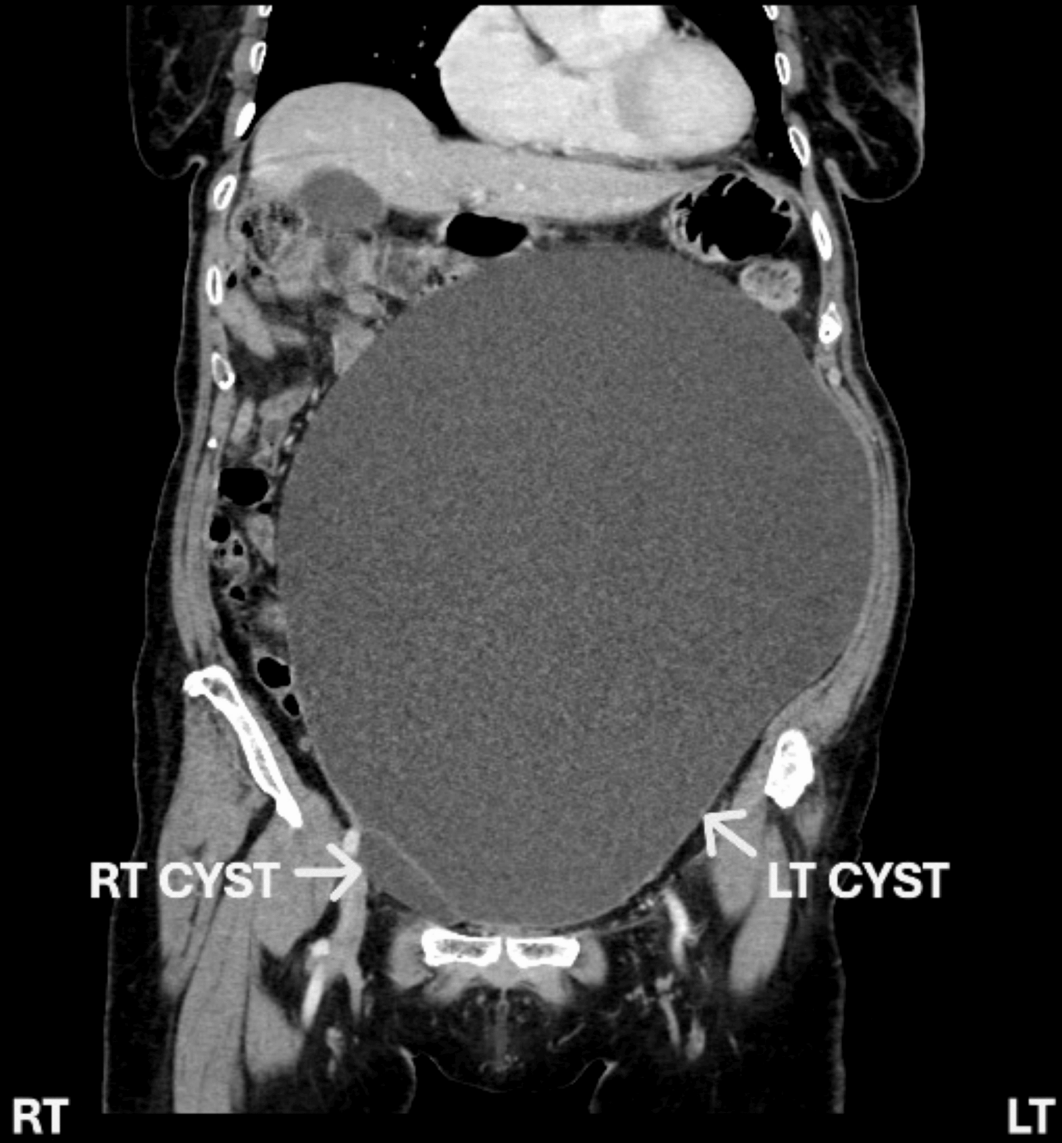
Contrast-enhanced abdominal computed tomography (coronal reconstruction) Demonstrating a large, well-defined cystic lesion occupying the majority of the abdominal cavity, the lesion has thin walls, homogeneous fluid attenuation, and lacks enhancing solid components. It is most likely arising from the left ovary. A smaller cystic lesion is also noted in the region of the right ovary.

From the gynecology history, the patient mentioned that she had undergone a Pap smear test 15 years ago, and mentioned a normal test with no signs of cervical inflammation or any other cervical pathology. Her menarche was at the age of 14; the menstrual cycle was every 30 days and lasted four days. The last menstrual period was recorded at the age of 53 years old. Obstetric history revealed that she had two vaginal deliveries with episiotomy. No abortions or miscarriages reported. Surgical history noted a surgical excision of a cyst formation in the left ovary at the age of 23. An examination of the abdomen showed a large, palpable mass that extended past the umbilicus, indicating a 34-week-old gravid uterus. Both the mesogastrium and the hypogastrium were tender. Peritoneal irritation was not evident. A large, firm, palpable, painless mass from the mesogastrium and hypogastrium with limited mobility was visible upon palpation. For further examination, we performed a contrast-enhanced CT scan and demonstrated a large, thin, well-defined cystic lesion with homogenous contents and no contrast enhancement measuring 32 x 28 x 15 cm, occupying the majority of the abdominal cavity, and a small cystic lesion from the right ovary. There was no observed lymphadenopathy or ascites at the CT scan examination (Figure 1).

As a result of her abdominal pain and discomfort, the patient desired admission to the gynecological clinic for a surgical operation. At her admission, the laboratory results showed no evidence of inflammation or increased values of tumor markers (Table 1). 

**Table 1:** Laboratory results N/A: not available; WBC: white blood cell; LYMPH: lymphocyte; HGB: hemoglobin; HCT: hematocrit; INR: international normalized ratio; aPTT: activated partial thromboplastin time; PLT: platelet; GLC: glucose; URE: urea; CRE: creatinine; K+: potassium; Na+: sodium; TPR: total protein; ALB: albumin; AST: aspartate transferase; ALT: alanine transaminase; GGT: gamma-glutamyl transferase; UA: uric acid; TSH: thyroid-stimulating hormone; FREE T3: free triiodothyronine; FREE T4: free thyroxine; CEA: carcinoembryonic antigen; AFP: alpha-fetoprotein; CA 19-9: cancer antigen 19-9; CA 125: cancer antigen 125; CA 15-3: cancer antigen 15-3

Parameter	Admission Day	Day 0 (Operation Day)	Day 1	Day 3	Day 5 (Discharge Day)	Follow-up (60 Days)	Reference Values
WBC	5.54 k/μL	15.19 k/μL	18.79 k/μL	10.10 k/μL	6.36 k/μL	10.2 k/μL	4-11 k/μL
Neutrophils	66.1 %	95.1 %	88.9 %	80.3 %	66.2 %	68.0 %	40-75%
LYMPH	24.9 %	2.8 %	5.7 %	13.5 %	24.2 %	28.0 %	20-45%
HBG	11.8 g/dl	11.1 g/dl	10.8 g/dl	10.3 g/dl	10.2 g/dl	12.5 g/dl	11.8-17.8 g/dl
HCT	36.4 %	35.0 %	33.9 %	32.3 %	31.8 %	36.9 %	36-52%
INR	1.07 sec	N/A	1.21 sec	N/A	N/A	N/A	0.8-1.2
aPTT	28.6 sec	N/A	29.2 sec	N/A	N/A	N/A	26-36 sec
PLT	207 k/μl	199 k/μl	224 k/μl	210 k/μl	242 k/μl	230 k/μl	140-450 k/μl
GLC	93 mg/dl	N/A	100 mg/dl	138 mg/dl	134 mg/dl	89 mg/dl	70-115 mg/dl
URE	30 mg/dl	N/A	26 mg/dl	16 mg/dl	35 mg/dl	28 mg/dl	10-50 mg/dl
CRE	0.75 mg/dl	N/A	0.67 mg/dl	0.66 mg/dl	0.66 mg/dl	0.80 mg/dl	0.5-1.1 mg/dl
K+	4.4 mmol/l	N/A	3.8 mmol/l	2.6 mmol/l	3.1 mmol/l	4 mmol/l	3.5-5.1 mmol/l
Na+	140 mmol/l	N/A	134 mmol/l	137 mmol/l	142 mmol/l	139 mmol/l	136-146 mmol/l
TPR	7.3 g/dl	N/A	6.3 g/dl	6.0 g/dl	6.1 g/dl	7.5 g/dl	6.2-8.4 g/dl
ALB	4.3 g/dl	N/A	3.7 g/dl	3.5 g/dl	3.5 g/dl	4.5 g/dl	3.5-5.1 g/dl
AST	26 U/L	N/A	23 U/L	27 U/L	39 U/L	24 U/L	5-33 U/L
ALT	16 IU/l	N/A	15 IU/l	23 IU/l	34 U/L	15 U/L	5-32 IU/l
GGT	13 IU/l	N/A	11 IU/l	31 IU/l	60 U/L	15 U/L	5-31 IU/L
UA	3.8 mg/dl	N/A	2.4 mg/dl	2.9 mg/dl	N/A	4.2 mg/dl	2.3-6.1 mg/dl
TSH	1.68 μIU/mL	N/A	N/A	N/A	N/A	1.70 μIU/mL	0.35-4.94 μIU/mL
FREE T3	2.63 pg/ml	N/A	N/A	N/A	N/A	2.40 pg/ml	1.88-3.18 pg/ml
FREE T4	0.86 ng/dl	N/A	N/A	N/A	N/A	0.90 ng/dl	0.70-1.48 ng/dl
CEA	2.28 ng/ml	N/A	N/A	<1.73 ng/ml	N/A	1.12 ng/ml	0-5 ng/ml
AFP	5.34 ng/ml	N/A	N/A	3.86 ng/ml	N/A	0.20 ng/ml	0.89-8.78 ng/ml
CA 19-9	3.97 U/ml	N/A	N/A	4.18 U/ml	N/A	0.9 U/ml	0-37 U/ml
CA 125	15.1 U/ml	N/A	N/A	28.9 U/ml	N/A	0.5 U/ml	0-35 U/ml
CA 15-3	20.6 U/ml	N/A	N/A	16.2 U/ml	N/A	0.22 U/ml	0-31.3 U/ml

**Figure 2:**
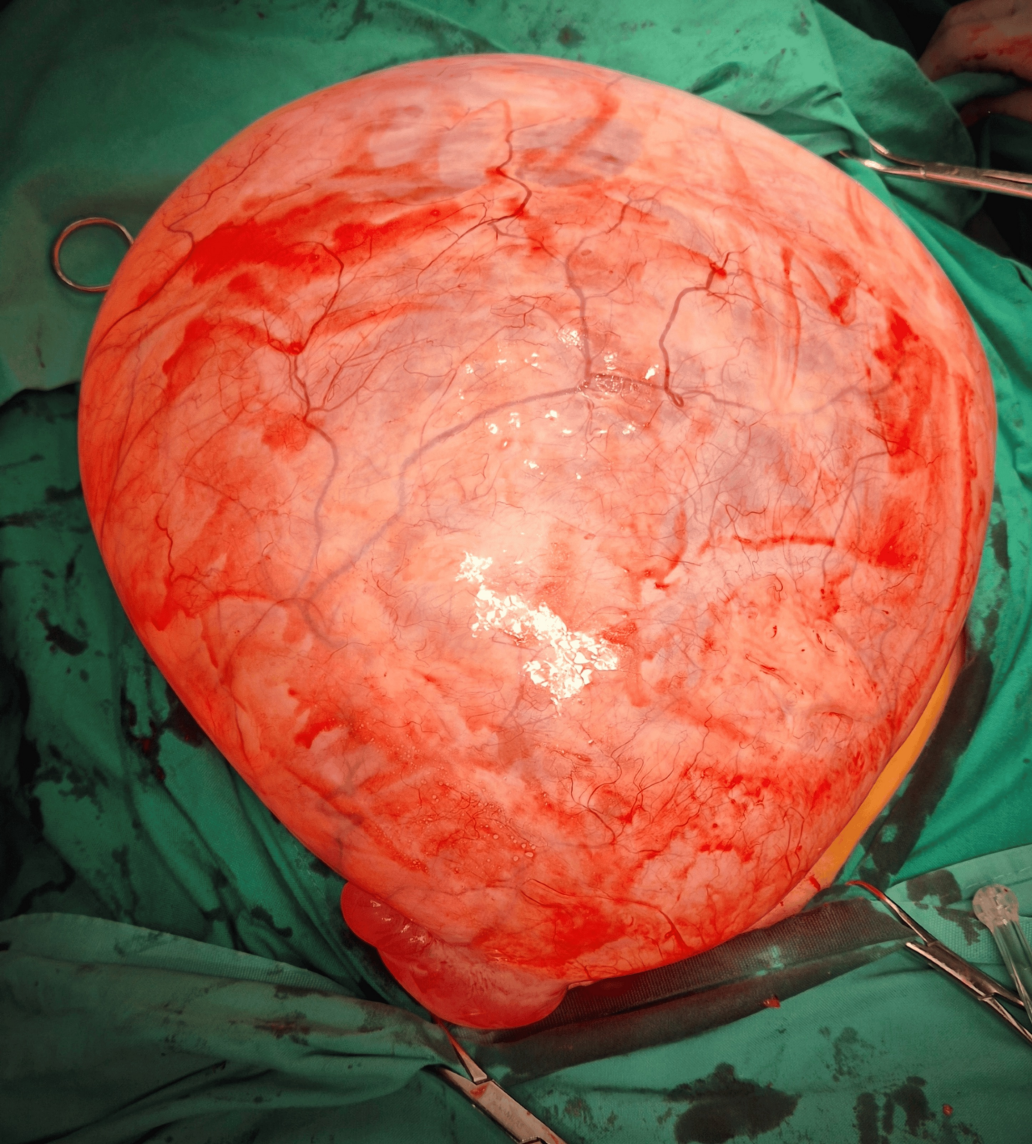
Macroscopic appearance of a giant benign serous cystadenoma during operation

In the operating room, the patient was positioned in a supine position and received general anesthesia. A midline incision was performed, and we identified a huge, giant cystic mass arising from the left ovary; we identified a small cyst at the right ovary (Figure 2). 

**Figure 3:**
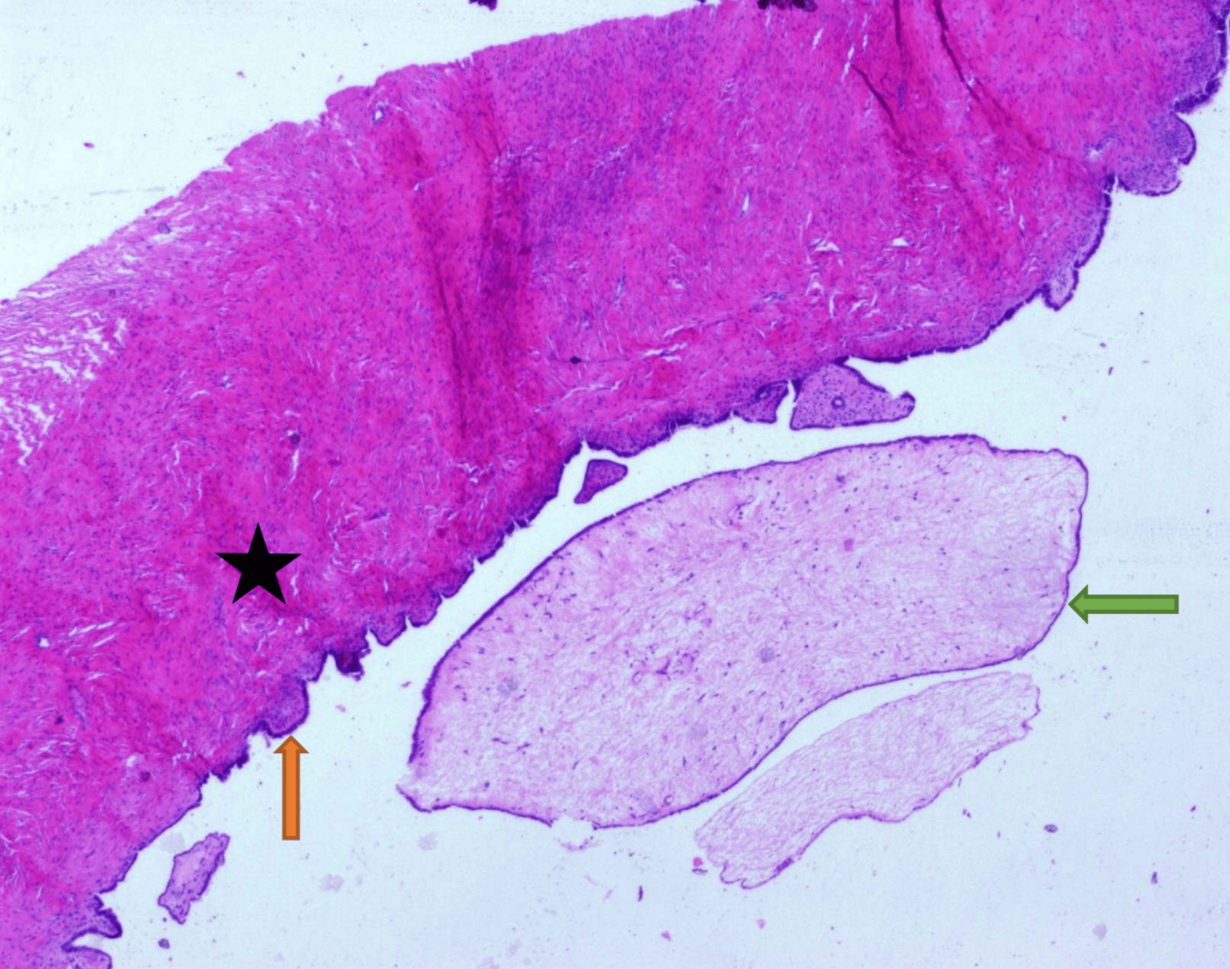
Histological section of the benign serous cystadenoma (H&E stain 10x) The cyst is lined by a single layer of columnar (orange arrow) or cuboidal epithelium (green arrow) with fibrous stroma (asterisk). H&E: histology stain of hematoxylin and eosin

The uterus appeared to be normal, and no free fluid was detected in the pouch of Douglas. We surgically performed a total hysterectomy with bilateral salpingo-oophorectomy (THBSO). Hemostasis was verified across the entire operative area. The abdominal wall layers were carefully repositioned in their proper anatomical sequence, and the closure of the skin was finalized with surgical staples. The giant left ovarian specimen weighed approximately 7.7 kg. And measured in diameter of 40 cm. The uterus, a giant left cyst formation, and the right small cyst were sent to the pathology department. Macroscopically, both cyst formations had a smooth surface. Upon incision, the giant cyst formation was lined by a single layer of columnar and cuboidal epithelium with fibrous stroma (Figure 3). 

Histopathology examination (HPE) confirmed a benign serous cystadenoma. After the operation, the patient was recovering well and was discharged from the gynecological unit on the fifth day. At the 60-day follow-up, no issues were noted.

## Discussion

Giant ovarian serous cysts affect women and teenagers globally, with symptoms including adnexal torsion and conditions similar to paraovarian cysts. Timely identification of pathology, including screening tools and operative fertility-sparing operations, is essential to therapy for such cyst masses [7,8]. Imaging results can complicate the differential diagnosis of giant adnexal cystic masses, which may overlap with various gynecologic and nongynecologic conditions. Important differential diagnoses include paraovarian cysts, mucinous cystadenoma, mature cystic teratoma, and others. Paraovarian cysts can mimic ovarian serous cystadenomas, necessitating intraoperative findings and histopathological analysis for accurate diagnosis. Adnexal masses in postmenopausal women carry an increased risk of neoplastic transformation, requiring assessment through serum tumor markers, imaging features, and clinical examination. While ultrasound is the primary imaging modality, CT and MRI provide critical details. Serum CA-125 is widely used to assess malignancy risk, with elevated levels indicating a higher risk of cancer. Recent research has highlighted the utility of HE4 and the Risk of Ovarian Malignancy Algorithm (ROMA) for improved differentiation of benign and malignant lesions [9,10]. Guleria et al. conducted a cohort study of 139,466 women with benign ovarian tumors, finding that those with benign ovarian serous tumors have a heightened long-term risk of developing subsequent borderline ovarian tumors (SIR 1.69, 95% CI 1.39-2.03), particularly in women under 40 years old [11]. A research study retrospectively reviewed pathology records and yielded that 53.4% of ovarian serous cystadenomas have a doubling time (DT) of growth of less than three years. At five years, only 15.1% have DT and no growth at a percentage of 31.5 [12]. Tjokroprawiro et al. reported a case with a postmenopausal woman, 67 years old, who suffered from a giant benign serous cystadenoma in the right ovary. This case resulted in those giant cysts creating complications like ovarian torsion, and it is an emergent situation for clinicians to act emergently [13]. A case with a postmenopausal woman who had been diagnosed with a benign serous cystadenoma yielded that cystadenomas appearing to be bilateral in the literature, possible to a percentage of 20-25, may create malignancy [14]. A case with a young patient described abdominal pain and discomfort, and underwent laparotomy for a benign serous cystadenoma. The authors of this case mention that these cysts' potential can evolve into a malignancy, a severe medical condition [15]. Another case of a 59-year-old postmenopausal woman referred to early detection via screening tools and clinical examination in early stages, improving the patient's postoperative complications [16]. 

Prasad et al. revealed that laparoscopic therapy for giant serous cystadenomas is a safe method, though caution is necessary if the cyst may have progressed to cancer and was undiagnosed prior to the laparoscopic operation [17]. A case of a 63-year-old woman with twisted ovarian cysts causing acute abdominal pain in her lower abdomen was presented by Ali et al., underscoring the importance of clinician awareness in minimizing morbidity and mortality [18]. Four cases of postmenopausal women with symptoms such as a palpable mass, lower abdominal pain, and abdominal distension underwent hysterectomy and surgical excision of giant cyst masses. The studies emphasize the importance of preoperative imaging tools and tumor markers to assess potential malignancy. Clinicians should be aware of the combination of cystadenomas and thecoma-fibroma, a rare pathology requiring urgent management. Additionally, intraoperative and postoperative vigilance is critical in the multidisciplinary management of high-risk cases to prevent severe complications [19-22]. All case studies are reported in Table 2. 

**Table 2:** Reported relevant case studies on giant benign serous cystadenoma RT: right ovary; LT: left ovary; CT scan: computed tomography scan; MRI: magnetic resonance imaging; TAHBSO: total abdominal hysterectomy with bilateral salpingo-oophorectomy

Author/Publication Year/Reference	Type of study	Number of Patients	Mean Age (Years)	Symptoms	Cyst Location	Cyst Size (cm)	Menopausal Status	Imaging Method	Surgical Operation	Pathology Report	Results
Tjokroprawiro et al. 2025 [13]	Case Report	1	67	Abdominal pain	RT and LT	RT: 30 cm, LT: 9.6 cm	Postmenopausal	CT scan	Bilateral salpingo-oophorectomy	RT: benign serous cystadenoma with mature teratoma, LT: serous cystadenoma and dermoid cyst	Ovarian torsion can happen in postmenopausal women with giant ovarian cysts, where persistent pain may indicate the condition and requires urgent treatment.
Agarwal et al. 2025 [14]	Case Report	1	63	Abdominal distension and discomfort	RT	RT: 18.5 cm	Postmenopausal	Transabdominal U/S	Laparotomy	RT: benign serous cystadenoma	Serous cystadenomas, particularly when bilateral, may conceal a malignancy risk of 20-25%.
Faroun et al. 2025 [15]	Case Report	1	12	Abdominal distension and left lower abdominal pain	LT	LT: 30 cm	Nulligravida	Transabdominal U/S and CT scan	Laparotomy	LT: benign serous cystadenoma	In pediatric patients, certain subtypes of serous cystadenomas can resemble malignancy.
Kelly et al. 2024 [16]	Case Report	1	59	Vomiting, lower abdominal pain, ureteral dilation, and hydronephrosis	RT	RT: 16 cm	Postmenopausal	CT scan	Laparotomy	RT: benign serous cystadenoma	Early detection of giant ovarian serous cystadenomas is critical for clinicians, as clinical examination and screening play vital roles in identifying these tumors at an early stage, improving patient outcomes and management strategies.
Prasad et al. 2023 [17]	Case Report	1	19	Lower abdominal pain, respiratory discomfort, and weight loss	LT	LT: 21 cm	Nulligravida	CT scan	Laparoscopy	LT: serous cystadenoma	Treatment for giant ovarian serous cystadenomas can be effectively done via laparoscopy, regardless of cyst size. Caution is advised if cancer has not been detected before with a laparoscopic procedure.
Ali et al. 2023 [18]	Case Report	1	63	Lower abdominal pain, abdominal fullness	RT	RT: 15 cm	Postmenopausal	Transabdominal U/S	Laparotomy	RT: benign serous cystadenoma	The twisted ovarian cyst can lead to an acute abdomen, necessitating clinician awareness to prevent morbidity and mortality.
Balhara et al. 2023 [19]	Case Report	1	53	Lower abdominal pain	LT	LT: 15 cm	Postmenopausal	MRI	Laparotomy	LT: cystadenoma	In postmenopausal women, cystadenomas are benign tumors, and a rare pathology can occur when there is a combination of thecoma-fibroma group and epithelial surface involvement.
Begum et al. 2021 [20]	Case Report with Literature Review	1	52	Palpable mass	LT	LT: 20 cm	Postmenopausal	TVS	TAHBSO	LT: serous cystadenoma	Tumor markers and imaging tools aid in diagnosing ovarian pathology. Clinicians must recognise severe postoperative complications such as pulmonary edema, venous pooling after intrabdominal mass resection, and abrupt hypotension from decreased venous return.
Mulita et al. 2020 [21]	Case Report	1	68	Massive abdominal distension and difficulty breathing	RT	RT: 34 cm	Postmenopausal	MRI	Laparotomy	RT: serous cystadenoma	Imaging tools are necessary for the diagnosis of pelvic giant masses accompanied by lower abdominal pain. In some instances, according to the literature, a giant mass with an elevated CA-125 tumor marker may be a benign condition.
Fatema et al. 2018 [22]	Case Report with Brief Literature Review	1	57	Palpable mass	LT	LT: 43 cm	Postmenopausal	Transabdominal ultrasound	TAHBSO	LT: serous cystadenoma	Strict intraoperative and postoperative vigilance is essential in the multidisciplinary management of high-risk cases to prevent severe complications.

The frequency of gynecological follow-up for ovarian cysts in postmenopausal women is determined by a number of factors, including the size of the cyst, imaging features, symptoms, and serum tumor markers. Simple ovarian cysts under 5 cm that are asymptomatic and have normal CA-125 levels can be evaluated and tested every four to six months. Due to the low risk of cancer, follow-up may be stopped if cysts stay stable or shrink in size after a year. On the other hand, because of the increased risk of cancer and possible complications, large cystic masses or symptomatic tumors require surgery. Optimizing outcomes for elderly patients requires multidisciplinary management and postoperative surveillance [23,24].

In our case study, a giant benign serous cystadenoma was identified with no potential for malignancy, and no postoperative complications were reported after 60 days. Future molecular and clinical studies are needed to enhance understanding and early diagnosis of these cyst formations affecting women of all age groups.

## Conclusions

Giant benign serous cystadenomas can affect women of all age groups and seem to be more commonly presented in postmenopausal women. The clinicians should consider in the differential diagnosis such huge abdominal masses that present nonspecific symptoms such as lower abdominal pain, pelvic pain, dyspnea, and abdominal discomfort. Our case demonstrates that this pathology is not ruled out by the normal tumor marker values. A precise preoperative diagnosis for this medical condition requires imaging screening and clinical examination. For the therapy, it is established that in young women, a laparoscopic procedure is more effective when it comes to patients' trauma and improving fertility results. For postmenopausal women, an operation option remains the TAHBSO. Studies have shown that this type of therapy has favorable outcomes. Generally, for such huge cystic masses, primary detection and prompt intervention are crucial in the reduction of complications and patient prognosis.

## References

[1] Sujatha VV, Babu SC (2009). Giant ovarian serous cystadenoma in a postmenopausal woman: a case report. Cases J.

[2] Cheng EJ, Kurman RJ, Wang M, Oldt R, Wang BG, Berman DM, Shih IeM (2004). Molecular genetic analysis of ovarian serous cystadenomas. Lab Invest.

[3] Limaiem F, Mlika M (2026). Ovarian cystadenoma. StatPearls [Internet].

[4] Kotis A, Karatapanis S, Papamargaritis V, Lisgos P, Sisamotos G (2011). A case of a large ovarian tumour. BMJ Case Rep.

[5] Liu X, Liu J, Chen L, Yang C, Hu Y, Liu Y (2024). Giant ovarian solid and cystic masses mixed with three types of tumors: a rare case report and literature review. Heliyon.

[6] Dey M, Pathak N (2011). Giant serous papillary cystadenoma. Med J Armed Forces India.

[7] Mpourazanis G, Aliri M, Papalexis P (2025). Giant paraovarian cysts in an adolescent female patient: a case report and literature review. Cureus.

[8] Korkontzelos I, Athanasiou G, Mpourazanis G (2026). Dermoid cyst with adnexal torsion presenting as acute appendicitis in a young premenstrual patient. Cureus.

[9] Carvalho JP, Moretti-Marques R, Filho AL (2020). Adnexal mass: diagnosis and management. Rev Bras Ginecol Obstet.

[10] Sadlecki P, Dejewska K, Domieracka P, Walentowicz-Sadlecka M (2025). Giant borderline ovarian tumours - review of the literature. Open Med (Wars).

[11] Guleria S, Jensen A, Kjær SK (2018). Risk of borderline ovarian tumors among women with benign ovarian tumors: a cohort study. Gynecol Oncol.

[12] Frederick RP, Patel AG, Young SW, Dahiya N, Patel MD (2021). Growth rate of ovarian serous cystadenomas and cystadenofibromas. J Ultrasound Med.

[13] Tjokroprawiro BA, Novitasari K, Ulhaq RA (2025). Torsion giant ovarian cysts in a postmenopausal woman with cervical cancer. Oxf Med Case Reports.

[14] Agarwal M, Jindal S (2025). Huge serous cystadenoma in a postmenopausal woman: a case report. IJRCOG.

[15] Faroun A, Gharbia BA, Sarahna A (2025). Coexistence of giant serous cystadenoma and hemorrhagic corpus luteal cyst in a 12-year-old: diagnostic and fertility-sparing surgical approach. BMC Pediatr.

[16] Kelly LP, Patel RB, Laan D (2024). Incidentally discovered giant benign ovarian serous cystadenoma in elective bariatric surgery. Cureus.

[17] Prasad I, Sinha S, Sinha U, Agarwal M (2023). Complete laparoscopic ovarian cystectomy of giant ovarian serous cystadenoma. Cureus.

[18] Ali S, Dhobale AV, Kalambe MA, Bankar NJ, Hatgaonkar AM (2023). Challenging diagnosis and management of an ovarian cyst torsion in a postmenopausal woman: a case report. Cureus.

[19] Balhara K, Mallya V, Khurana N, Tempe A (2023). Coexisting ovarian serous cystadenoma with fibroma: a very unusual combination. J Cancer Res Ther.

[20] Begum DA, Fatema S (2021). Giant ovarian cyst adenoma in a postmenopausal woman: a case report with literature review. Gynecol Obstet Case Rep.

[21] Mulita F, Tavlas P, Maroulis I (2020). A giant ovarian mass in a 68-year-old female with persistent abdominal pain and elevated serum CA-125 level. Prz Menopauzalny.

[22] Fatema N, Mubarak Al Badi M (2018). A postmenopausal woman with giant ovarian serous cyst adenoma: a case report with brief literature review. Case Rep Obstet Gynecol.

[23] RCOG Green-top Guideline No. 34 (2025). Royal College of Radiologists. Ovarian cysts found incidentally in postmenopausal patients: follow-up. iRefer Guideline OG15. The Management of Ovarian Cysts in Postmenopausal Women.

[24] Valentin L, Ameye L, Franchi D (2013). Risk of malignancy in unilocular cysts: a study of 1148 adnexal masses classified as unilocular cysts at transvaginal ultrasound and review of the literature. Ultrasound Obstet Gynecol.

